# Acute toxicity analysis of an inhibitor of BCL2, Disarib, in rats

**DOI:** 10.1038/s41598-021-89387-x

**Published:** 2021-05-11

**Authors:** Shivangi Sharma, Kontham Kulangara Varsha, Ujjayinee Ray, Humaira Siddiqua, Anjana Elizabeth Jose, Sridhar Muninarasimaiah, Sathees C. Raghavan, Bibha Choudhary

**Affiliations:** 1 Institute of Bioinformatics and Applied Biotechnology, Electronics City, Bangalore, 560100 India; 2 Department of Biochemistry, Indian Institute of Science, Bangalore, 560012 India; 3 Manipal Academy of Higher Education, Manipal, Karnataka 576104 India

**Keywords:** Biotechnology, Drug discovery

## Abstract

Apoptosis or programmed cell death is a highly regulated process, which eliminates unwanted and damaged cells. Inhibition of apoptosis is a hallmark of cancer cells. BCL2 family proteins are known to play a vital role in the regulation of apoptosis. Overexpression of BCL2, an antiapoptotic protein, provides the advantage of prolonged survival to cancer cells. Over the years, several BCL2 inhibitors have been investigated extensively for their anticancer potential. However, most of them were abolished before clinical use due to their side effects. Previously, we had identified and characterized a novel BCL2 inhibitor, Disarib, with the potential to eliminate tumor cells in a BCL2 specific manner leading to reduction in tumor burden in multiple mouse models. Notably, a head-to-head comparison of Disarib to ABT199, the only FDA approved BCL2 inhibitor revealed that Disarib is as potent as ABT199. Recent studies using mice revealed that Disarib did not invoke significant side effects in mice. In the present study, we have investigated the acute toxicity of Disarib in Wistar rats. The bioavailability studies following exposure of Disarib in Wistar rats revealed its maximum availability in serum at 24 h following oral administration. Acute toxicity analysis revealed that even a dose as high as 2000 mg/kg of Disarib did not cause significant toxicity in rats. There was no significant variation in blood parameters or kidney and liver functions following administration of Disarib. Histological analysis of different tissues from Disarib treated groups revealed standard architecture with no observable cellular damage. Importantly, exposure to Diasrib did not result in genotoxicity as determined by micronucleus assay. Further, solubility assays revealed that besides DMSO, Disarib is also soluble in alcohol. While the high acidic condition can increase the solubility of Disarib, even a lower percentage of alcohol with acidic conditions can improve its solubility. Thus, the toxicological profile in the current study revealed no significant side effects when Disarib was administered orally to rats.

## Introduction

Impaired apoptosis is one of the key strategies of cancer cells for survival^1,2^. BCL2 is an antiapoptotic protein known to be overexpressed in several cancers^1,3–5^. Since BCL2 family proteins have a vital role in the regulation of apoptosis, over the years, several strategies have been used to target BCL2 for anticancer therapies^3,4,6–9^. The use of small molecule inhibitors for disrupting the interaction between BCL2 with its proapoptotic partners (BAK/BAX) has become popular^6,10–12^. Several small molecule inhibitors that target BCL2 have been investigated extensively for their anticancer potential, such as Gossypol, YC137, HA14.1, TW37, Chelerythrine, ABT737, AT101, Obatoclax, ABT263 and ABT199^11,13–16^. Unfortunately, most BCL2 inhibitors failed in clinical trials due to their pan activity^17,18^. Some of the BCL2 inhibitors, such as ABT737, ABT263, and ABT199, had efficient clinical relevance; however, ABT737 and ABT263 were eliminated due to dose-limiting toxicities, including neutropenia and thrombocytopenia^4,19^. Only one BCL2 inhibitor, ABT199 (Venetoclax), has been approved by the Food and Drug Administration (FDA) to treat Chronic Lymphocytic Leukemia or Small Lymphocytic Lymphoma and Acute Myeloid Leukemia^6,11,20^.

Previously, we reported the identification of a novel BCL2 inhibitor, Disarib, which efficiently eradicated cancer cells with high BCL2 levels^21^. We showed that Disarib administration via intraperitoneal route in mice led to efficient tumor regression comparable to that of ABT199, without causing significant side effects^21–23^. Since ABT199 is the only FDA approved drug for anti-tumor effects, we were interested in pursuing further studies to facilitate clinical translation of Disarib. Importantly, toxicological studies of Disarib in mice revealed LD_50_ of > 2000 mg/kg when administered via oral route^22^. Moreover, Disarib specifically interacted with BCL2, but not with other antiapoptotic proteins such as BCL-xL, which is one of the reasons for its platelet sparing property, as BCL-xL is the primary survival factor for platelets^12,23^. We also observed that treatment of Disarib resulted in disruption of the interaction of BCL2 with its proapoptotic partner BAK. However, it did not disrupt the interaction of BCL2 with BAX or with other members of the pro-apoptotic family, which finally resulted in the activation of the intrinsic pathway of apoptosis followed by cell death^12^, suggesting that Disarib is a promising BCL2 inhibitor and thus underlining the need for further studies.

Based on the guidelines by Central Drugs Standard Control Organization (CDSCO), India on the preclinical evaluation of any chemical compound intended for human use, in our previous study we had performed single dose toxicity studies of Disarib by oral administration in mice^22^. Results revealed that even the highest dose of Disarib, 2000 mg/kg, did not cause any visible toxicity in mice. The behavior of treated animals and food and water consumption were comparable to that of control animals. Moreover, oral administration in mice led to significant reduction in tumor progression in multiple mice tumor models. Further, oral Disarib treatment did not affect the platelet count even at the highest dose, unlike several other BCL2 inhibitors^7,22^.

In the present study we have reported the preclinical acute toxicity, pharmacokinetics, and pharmacodynamics of BCL2 inhibitor, Disarib, in Wistar rats. As per CDSCO, India and FDA, USA guidelines, systemic toxicity in mice should be confirmed in another rodent species (preferably rats) in order to establish linear relationship between toxicity and body surface area, so as to further determine the starting dose for Phase I trial. Our study showed that oral administration of Disarib in rats did not lead to any significant behavioral or body weight changes. Analysis of biochemical, histological, blood parameters, and genotoxic effects revealed that even at the highest dose (limiting dose) of Disarib, there was no significant toxic effect in Wistar rats. Importantly, HPLC studies revealed bioavailability up to 24 h following oral administration of Disarib. Thus, the toxicological profile described in this study underlines the potential clinical relevance of the new BCL2 inhibitor.

## Results

### Disarib is soluble in DMSO and alcohol

**Figure 1:**
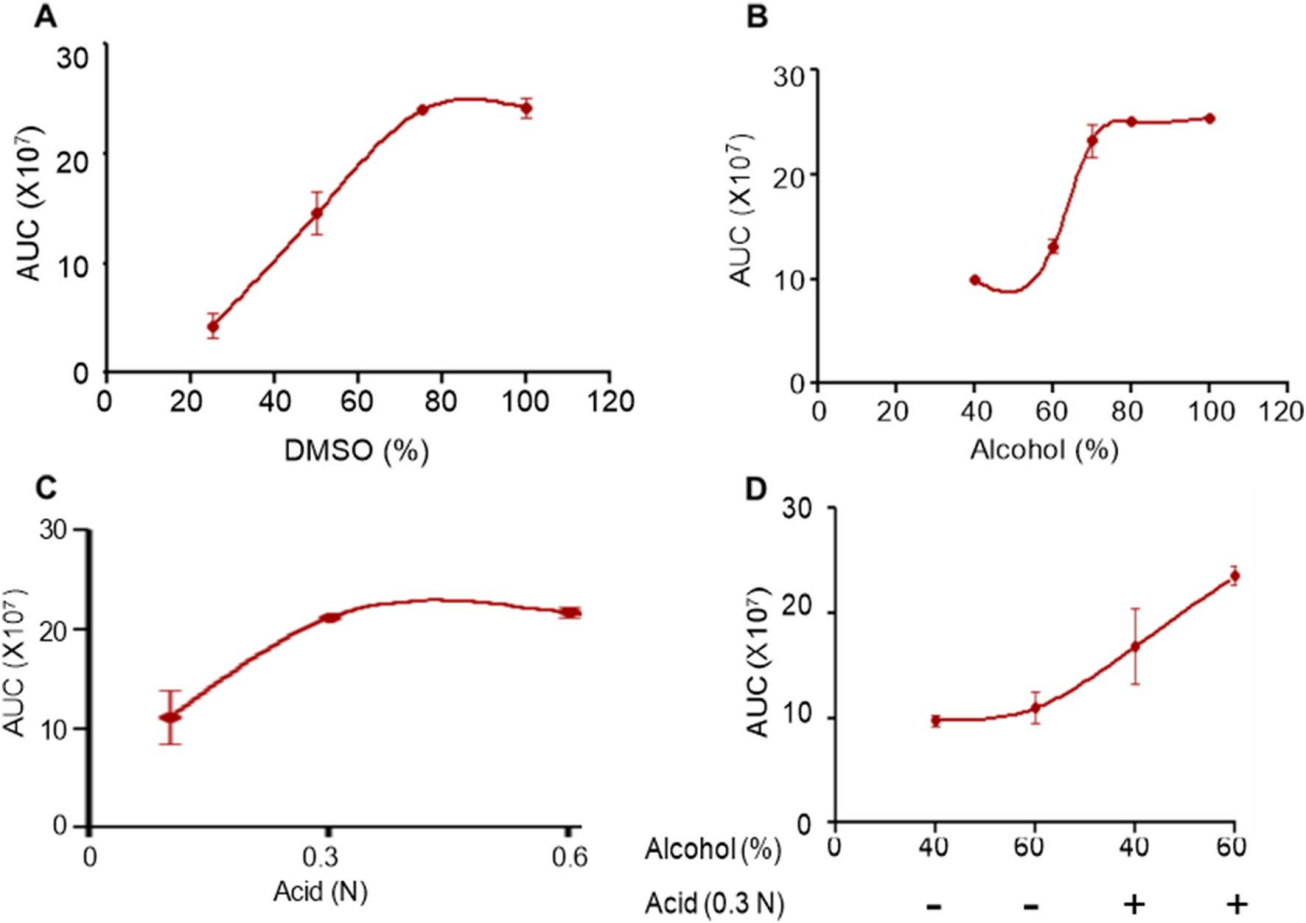
Evaluation of solubility of Disarib in different solvents. (**A–C**) Solubility of Disarib in different concentration of DMSO (**A**), alcohol (**B**) and HCl (**C**). Concentrations used were 25, 50, 70 and 100% for DMSO, 40, 60, 70, 80, and 100% for alcohol and 0.1, 0.3 and 0.6 N for HCl. (**D**) Analysis of solubility in alcohol (40, 60%) in combination with HCl. In each panel, HPLC analyses were performed at three independent times at 232 nm wavelength. The area under the curve (AUC) was calculated to obtain the solubility of Disarib.

It is crucial to evaluate the solubility potential of drugs in their early stage of development. Drug activity can be masked by low solubility^24^, leading to reduced absorption^25^. The solubility of Disarib was investigated by dissolving a fixed quantity of Disarib (100 µM) in increasing concentrations of DMSO, alcohol, and HCl (Fig. 1). Solubility potential was studied by HPLC analysis. Results showed that complete dissolution of Disarib was obtained with > 75% DMSO (Fig. 1A) and alcohol (75% or above) (Fig. 1B). Disarib was soluble only in higher acidic conditions such as 0.3 N HCl (Fig. 1C) when increasing acidic conditions were used. However, in combination with alcohol and acids, Disarib was soluble in 40% alcohol and 0.3 and 0.6 N HCl (Fig. 1D).

### Disarib is stable when dissolved in PBS

**Figure 2:**
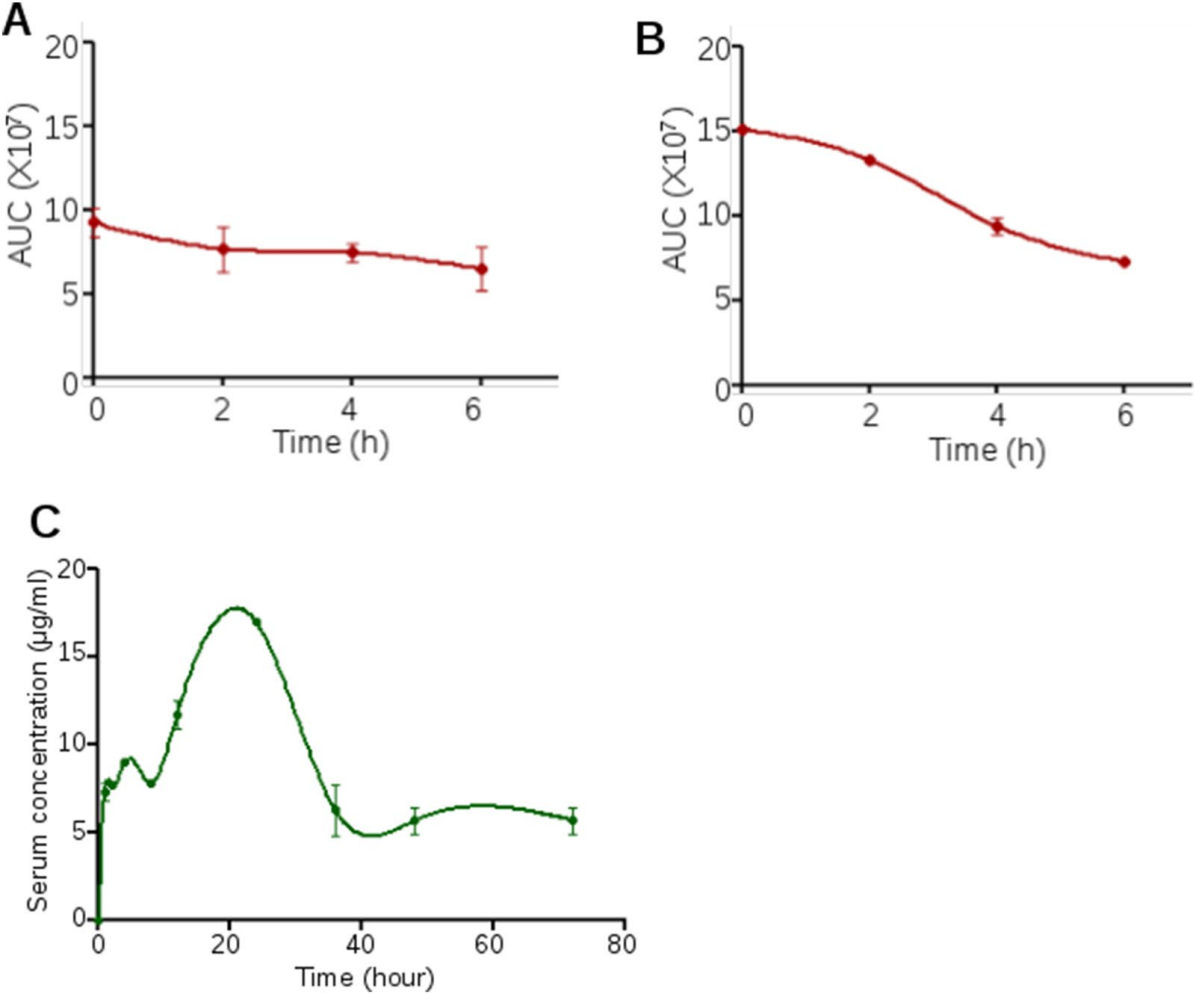
In vitro stability and in vivo bioavailability of Disarib in rats. (**A**,**B**) Stability analysis of Disarib at different time points (0, 2, 4 and 6 h) in PBS (**A**) and plasma (**B**). (**C**) In vivo bioavailability analysis of Disarib in the serum of Wistar rats at 0.25, 0.50, 1, 2, 4, 6, 8, 10, 12, 24, 48, 72 h after treatment. Two animals each were treated with Disarib orally and sera collected at each time points were subjected to HPLC analysis. The area under the curve (AUC) was calculated to obtain bioavailability. HPLC analysis were done at 232 nm wavelength.

The compound's stability plays an essential role in the early stage of drug development^26,27^. It will also help rationalize the dose of a compound used for clinical trials^24,25^. The stability of Disarib in plasma and PBS was investigated by dissolving Disarib (54.76 mg/ml) in either PBS or 80% plasma (diluted in PBS), and HPLC analysis was performed at different time points to evaluate the reduction in Disarib peak. Our results revealed that while Disarib was stable in PBS up to 6 h (Fig. 2A)*,* the stability of Disarib gradually decreased in plasma with increasing time (Fig. 2B).

### Pharmacokinetic profile of Disarib revealed maximum bioavailability at 24 h post-administration in rats

Investigating the pharmacokinetic profile of a compound can minimize the time taken for drug development as it eases the way for further early clinical trials^24^. In our previous study on intraperitoneal administration of Disarib (10 mg/kg) in mice maximum circulating concentration (Cmax) was obtained at 30 min, suggesting faster absorption in vivo^23^. We have also reported a t_1/2_ of 2.3 h and a clearance rate (CL) of 6.3 ml/min/kg for Disarib in mice. In the present study, serum samples were collected from the Wistar rats at different time points (0.25, 0.5, 1, 2, 4, 6, 8, 12, 24, 48 and 72 h) after oral Disarib treatment. Since oral route is the preferred route of administration for intended clinical use, the pharmacokinetics was determined upon Disarib administration (50 mg/kg b. wt) via oral route. The concentration of Disarib in serum was determined by calculating the area under the curve (AUC) using HPLC LabSolutions software. HPLC analysis post Disarib oral administration revealed that the maximum concentration of Disarib (Cmax) was ~ 18 μg/ml, time to Cmax (Tmax) was 24 h, and t_1/2_ was determined as 36 h in the serum of rats (Fig. 2C). Moreover, clearance rate and steady state volume of distribution (Vss) were estimated to be 0.44 ml/min/kg and 4.16 l/kg based on the rate of drug elimination and amount of drug in the plasma in equilibrium condition.

### Acute toxicity studies in rat revealed no visible toxicity following Disarib treatment

**Figure 3:**
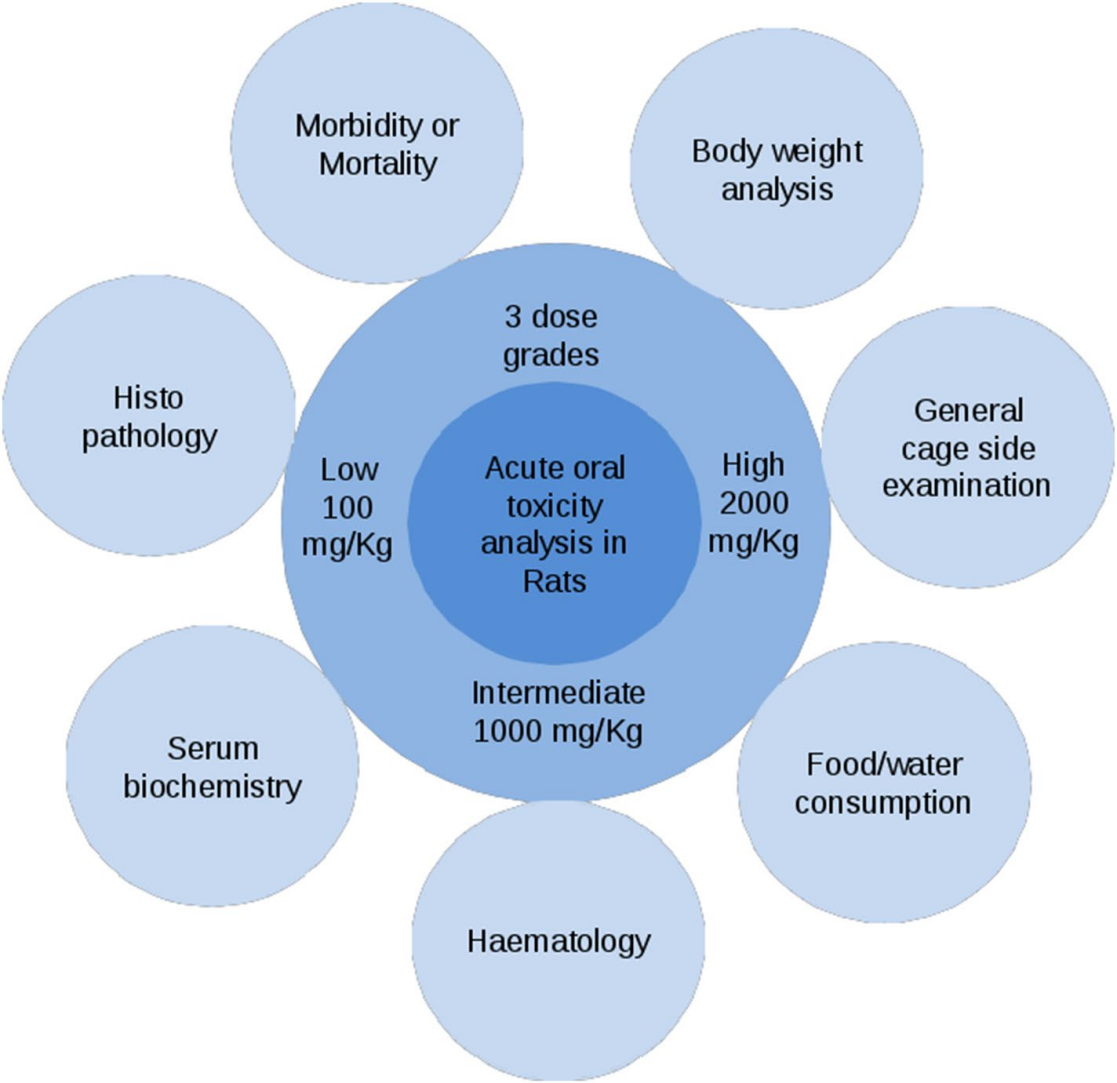
Schematic representation showing route followed for acute dose toxicity analysis following Disarib treatment in Wistar rats. 100, 1000 and 2000 mg/kg dose grades represents low, medium, and higher dose, respectively. Mortality, body weight, general cage side examinations, food and water consumption were analyzed everyday during the experiment. Haematology, serum biochemistry, and histopathology were analyzed after 14 days of dose administration.

To investigate toxic effects following administration of Disarib in a rodent model system, Wistar rats were selected for the present study based on the guidelines from CDSCO, India (Fig. 3). Previously, acute toxicity studies in mice did not reveal any significant toxic effects^22^. In the current study, three different dose ranges, low (100 mg/kg), medium (1000 mg/kg), and high (2000 mg/kg), were selected. Disarib was orally administered (n = 5) after preparing a solution using carboxymethyl cellulose, which also served as vehicle control.

Treated groups of rats and control animals were observed for mortality up to 14 days after Disarib oral administration as per the guidelines of CDSCO, India. All the experimental animals were alive throughout the experiment, and importantly, they were as healthy as control animals. Experimental animals were daily examined for signs of toxicity caused by oral administration of Disarib. Interestingly, we observed that all parameters analyzed, such as behavior, appearance, lachrymation, changes in locomotion, hair, and skin, were comparable to the vehicle control group.

**Figure 4:**
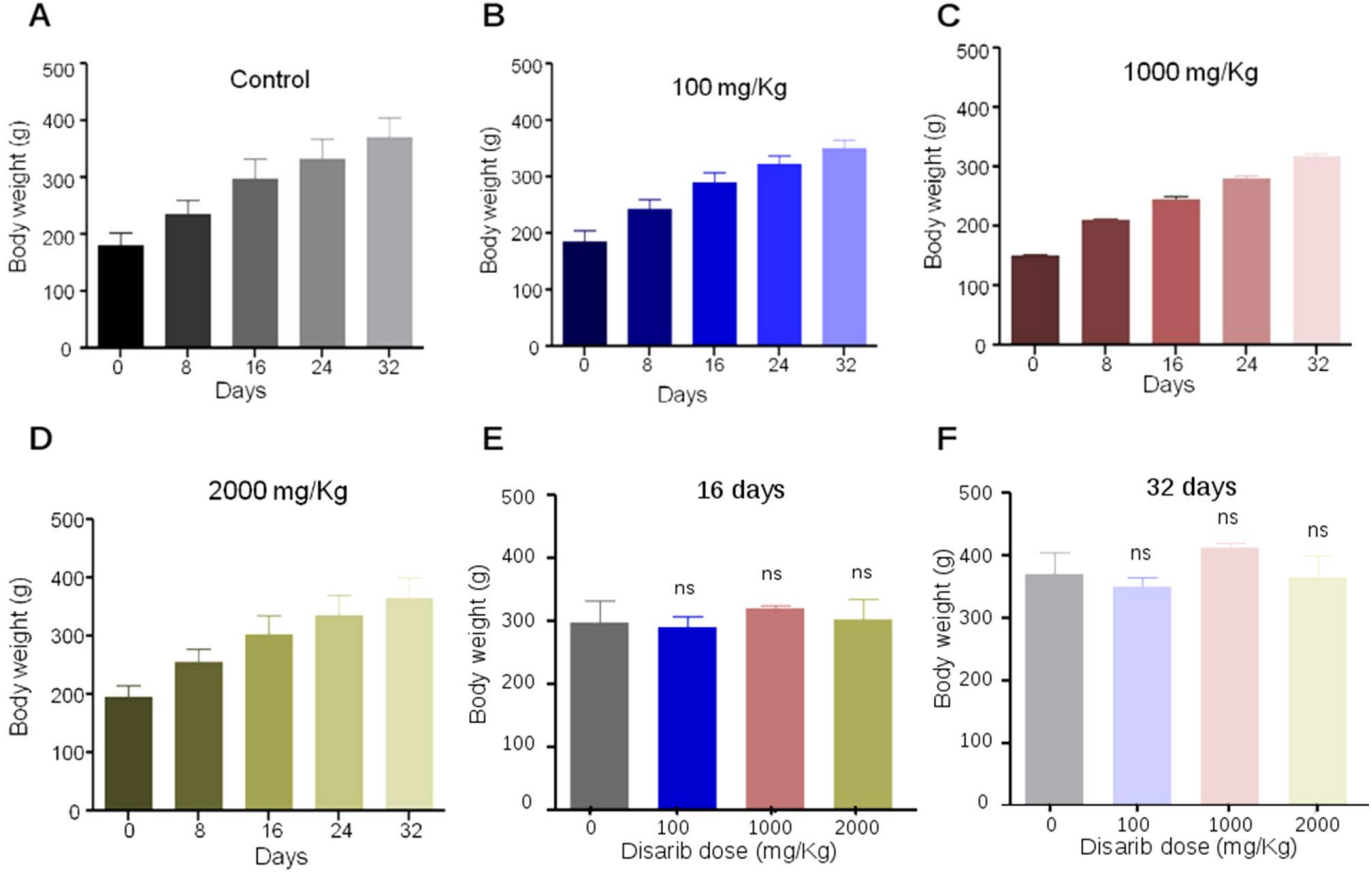
Single-dose toxicity studies of Disarib in Wistar rats. (**A**–**D**) Body weight analysis of rats on 0, 8, 16, 24, 32 days after oral administration of vehicle control (carboxymethyl cellulose) (**A**), low dose (100 mg/kg) (**B**), intermediate dose (1000 mg/kg) (**C**) and high dose (2000 mg/kg) (**D**) of Disarib. (**E**,**F**) Comparison of change in body weight among control and treated groups after 16 days (**E**) and 32 days (**F**). Five rats per group were used for the study. Error bars denote mean + SEM (*ns* not significant).

We observed that even at the highest dose tested (limiting dose of 2000 mg/kg b. wt.) Disarib did not cause mortality or any visible sign of toxicity in animals. In the absence of Disarib induced mortality in the highest dose group, the LD_50_ for oral dose of Disarib in Wistar rats was determined as > 2000 mg/kg b. wt, which was consistent with the LD_50_ determined in mice^22^. Maximum Tolerated Dose (MTD) was estimated to be > 2000 mg/kg since the animals did not show any observable body weight changes. Further, to investigate the impact of Disarib on body weight, rats were weighed periodically every 4 days up to 32 days, from the day of administration of the compound (Fig. 4). There was a gradual increase in body weight of the animals, both in control and treated groups (Fig. 4A–F). Further, evaluation of the effect of Disarib on food and water ingestion was assessed daily. The results revealed no difference in eating and drinking habits in treated groups and control animals, suggesting no alteration in food and water consumption following treatment of Disarib.

### Haematological studies show normal blood parameters following Disarib treatment in rats

**Figure 5:**
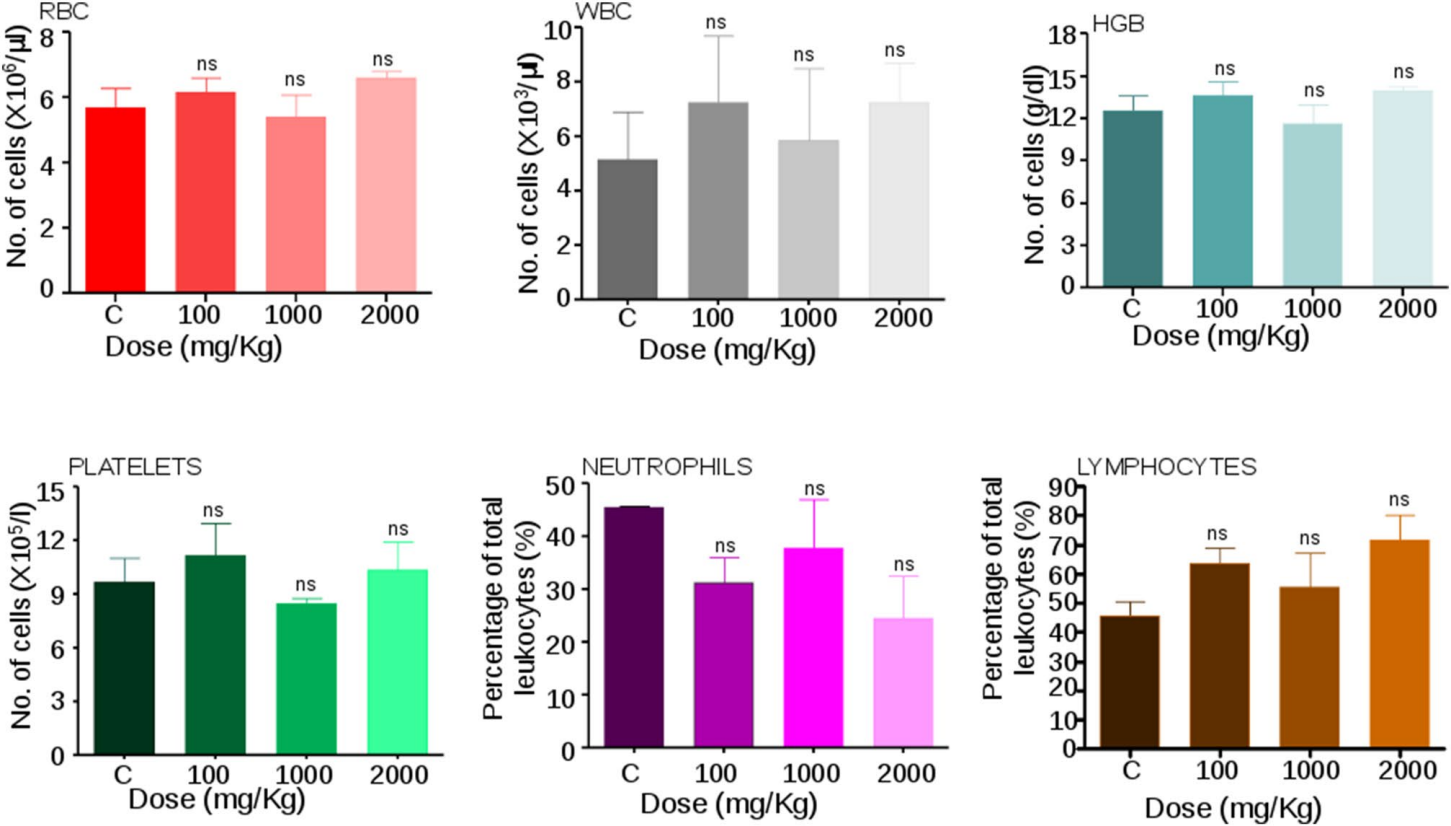
Blood parameter analysis following oral administration of Disarib in rats. Analysis of red blood corpuscles (RBC), white blood corpuscle (WBC), haemoglobin (HGB), Platelets, Neutrophils, Lymphocytes count among control and Disarib treated groups (100, 1000, 2000 mg/kg b. wt) after 14 days of dose administration. Error bars denote mean + SEM (*ns* not significant).

Blood was collected by heart puncture from treated and control Wistar rats after the 14th day of administration of Disarib in anticoagulant coated vials, and total blood count was evaluated. Results showed no significant difference in the total number of RBC and HGB content between control and treated groups, and the values were within the normal range (Fig. 5)^28–30^. The decrease in platelet count upon higher doses is commonly reported for most BCL2 inhibitors^4,19^. Although a marginal variation in platelet count was observed in the case of 1000 mg/kg b. wt. Disarib treated group; such a difference was not observed in lower and higher dose treated groups (100 and 2000 mg/kg, respectively) (Fig. 5). Therefore, these results suggested that Disarib treatment in rats did not result in dose-limiting toxicity such as thrombocytopenia (Fig. 5). The analysis also revealed that there was no significant difference in WBC counts between control and treated groups. Further, no significant difference was observed between the neutrophils and lymphocytes count in control and treated animals (Fig. 5). Although there were some variations among different groups and animals within the group, all the values were within the normal physiological range (Fig. 5)^28,30^. Analyses of PCV, MCV, MCH, MCHC, eosinophils, and monocytes suggested no toxic effects caused by Disarib as all these parameters were comparable to that of the control group (Table 1).

**Table 1:** Analyses of blood parameters after 14 days of oral administration of Disarib in Wistar rats.

Haematology	Vehicle control		100 mg/kg		1000 mg/kg		2000 mg/kg	
	a	b	a	b	a	b	a	b
Eosinophils (%)	1.2	1.3	1.0	1.2	1.0	1.2	1.0	1.1
Monocytes (%)	5.1	3.0	3.5	4.2	3.2	8.1	2.4	3.0
PCV (%)	37.8	39.8	40.0	38.4	40.2	36.9	41.2	43.3
MCV (fl)	62.4	76.8	60.2	66.6	65.7	78.6	63.7	63.3
MCH (pg)	17.2	21.9	21.9	22.0	21.2	21.9	21.2	20.7
MCHC (%)	33.7	28.6	36.5	33.0	32.3	27.9	33.4	43.3

Blood parameter analysis was done after 14 days of Disarib administration (0, 100, 1000, 2000 mg/kg b. wt.) by oral route. ‘a’ and ‘b’ are indicative of two different animals of the same group used for the study.

*PCV* packed cell volume, *MCV* mean cell volume, *MCH* mean corpuscular haemoglobin, *MCHC* mean corpuscular haemoglobin concentration.

### Administration of Disarib did not affect hepatic or renal functions

**Figure 6:**
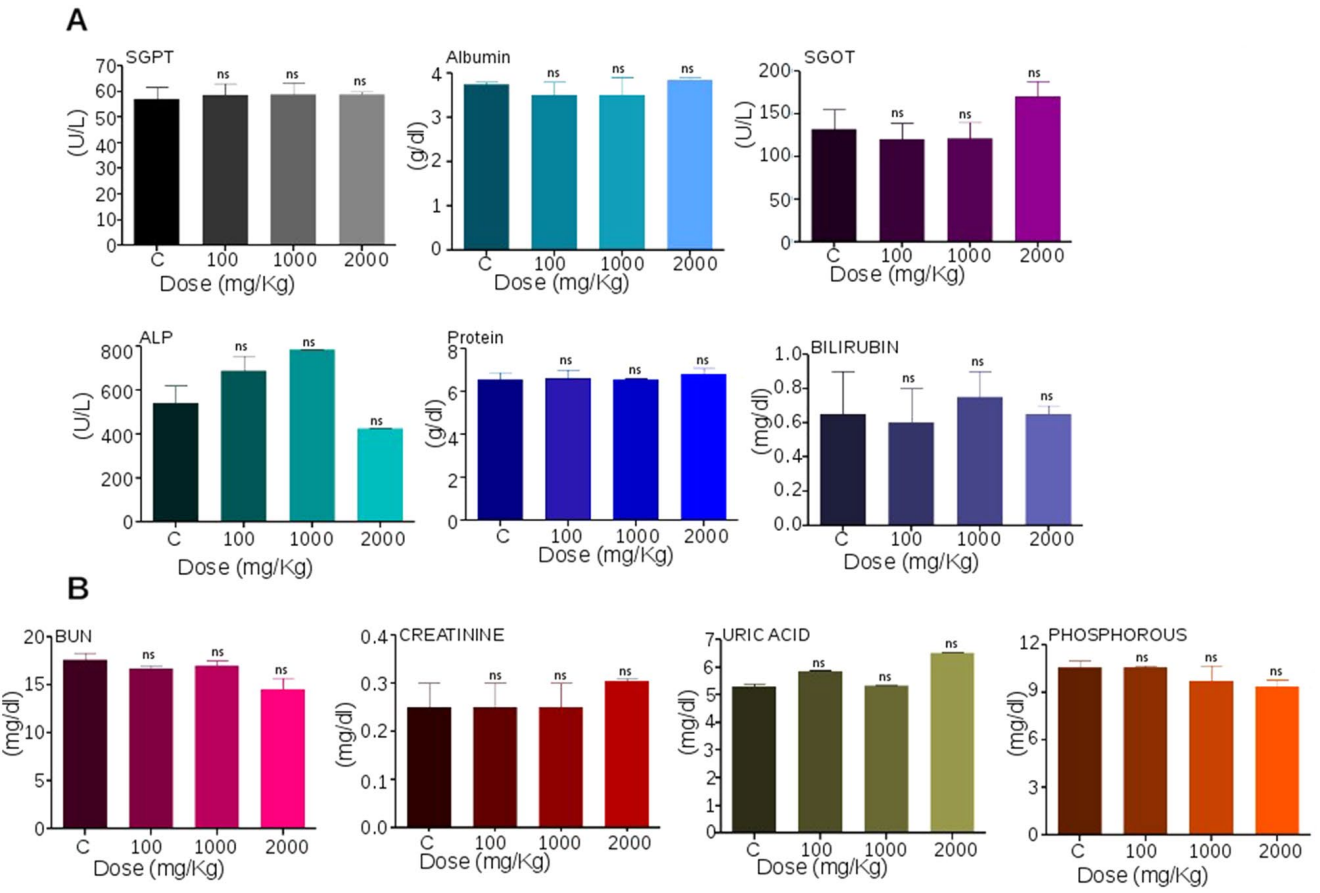
Analysis of liver and kidney function following treatment with Disarib in rats. (**A**) Analysis of serum parameters for liver function among control and different Disarib treated rats (100, 1000, 2000 mg/kg b. wt.) after 14 days of dose administration. *SGPT* serum glutamic pyruvic transaminase, *SGOT* serum glutamic-oxaloacetic transaminase, *ALP* alkaline phosphatase. (**B**) Analysis of serum parameters for kidney function among control and different doses of Disarib (100, 1000, 2000 mg/kg b. wt.) after 14 days of dose administration. *BUN* Blood urea nitrogen. Error bars denote mean + SEM (*ns* not significant).

After acute dose administration of Disarib in rats, serum was collected 14 days post treatment and analyzed for factors that indicate kidney and liver functions. Hepatic function tests for SGPT, ALP, SGOT, total protein, Albumin, and Bilirubin suggested that Disarib did not cause any adverse effect on liver function (Fig. 6A). Although, ALP levels observed were higher in 100 and 1000 mg/kg Disarib treated groups compared to control, the increase was limited and was not significant. Moreover, the group treated with 2000 mg/kg Disarib showed an ALP level similar to that of the control (Fig. 6A)^28,30,31^. Consistent with this, the level of SGOT was marginally higher in the maximal dose treated group; however, the increase was within the normal range and was not significant compared to control^28^ (Fig. 6A). Renal function analysis showed that BUN levels, Creatinine, Phosphorous, and Uric acid did not have any appreciable variation between treated and control groups (Fig. 6B). Thus, the above results suggested that tested doses of Disarib, including the highest (2000 mg/kg), showed neither mortality nor deviation from normal hepatic and renal function.

### Histopathological analysis does not reveal a significant change in the structure of tissues

**Figure 7:**
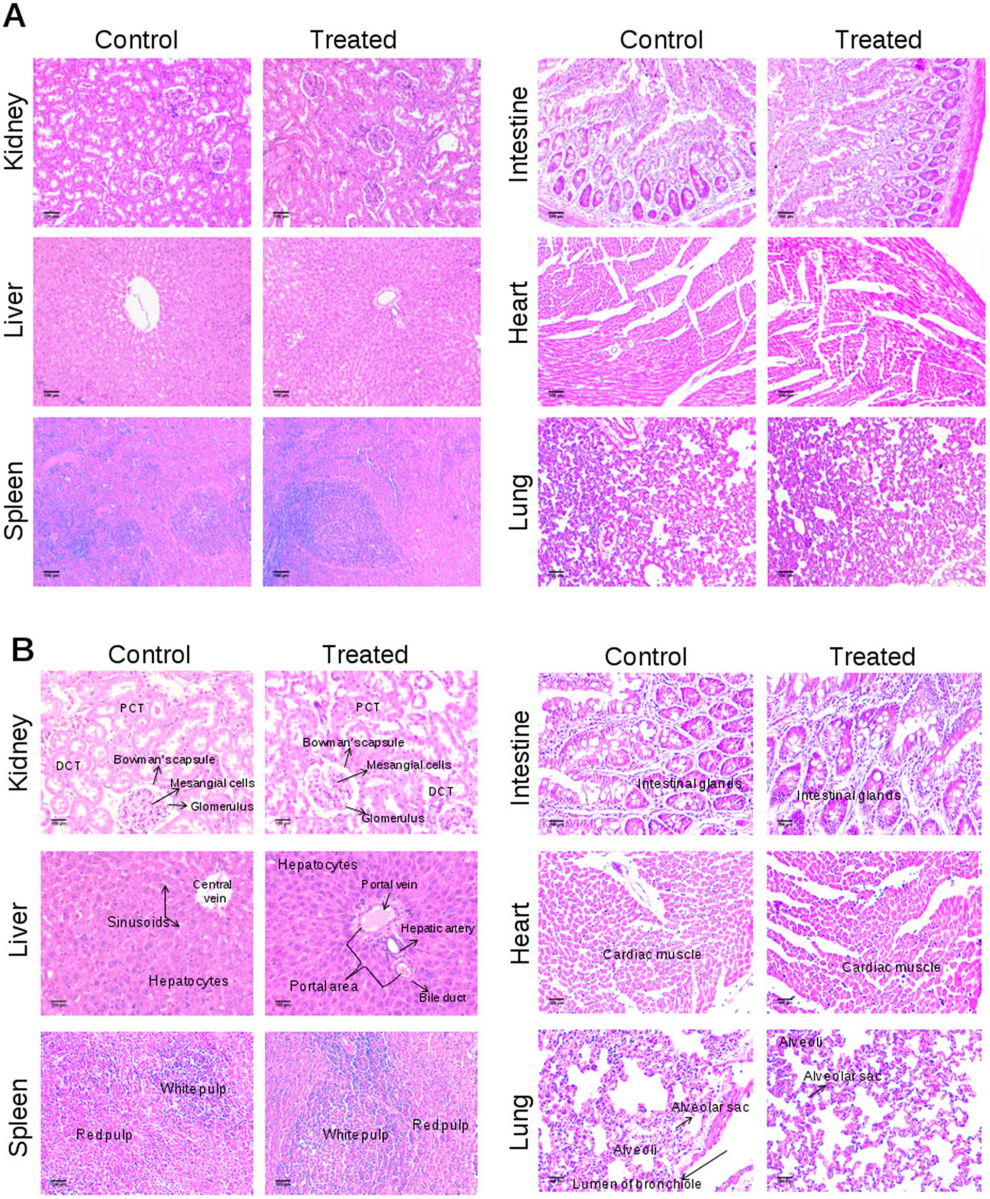
Histopathological analyses of different tissues of rat following administration of Disarib. (**A**,**B**) HE staining of tissues from control and 2000 mg/kg Disarib treated rats. Images shown are with a magnification of 10 × (**A**) or 20 × (**B**). *DCT* distal convoluted tubule, *PCT* proximal convoluted tubule. Scale bar 100 µm.

Since we did not observe any significant difference in blood and serum parameters between control and Disarib treated groups of rats, we performed histological analysis of liver, kidney, intestine, spleen, lung, and heart following Disarib administration to check for morphological changes. Results showed no cellular change in lobular liver texture, hepatocytes and sinusoidal spaces between control and treated animals (Fig. 7A,B). The kidneys of control and treated groups of animals showed a normal distribution of Malpighian corpuscles. No glomerular dilation and tubular necrosis were seen in the case of Disarib treated tissues (Fig. 7A,B). Histology of vital organs such as lungs and heart did not show difference in the structure of alveoli and cardiac muscles, respectively, between control and treated groups. Intact cardiac muscle cells in heart and intact alveoli and bronchiole structure in lungs were observed in control and treated groups (Fig. 7A,B). Normal structure and organization of the intestine were seen after treatment of Disarib (Fig. 7A,B). Similarly, no difference in spleen architecture was observed when the histological evaluation was performed (Fig. 7A,B). Thus, histological analyses and other studies revealed that the Disarib treatment did not cause any toxicity in rats.

### Disarb does not induce genotoxicity in rats

**Figure 8:**
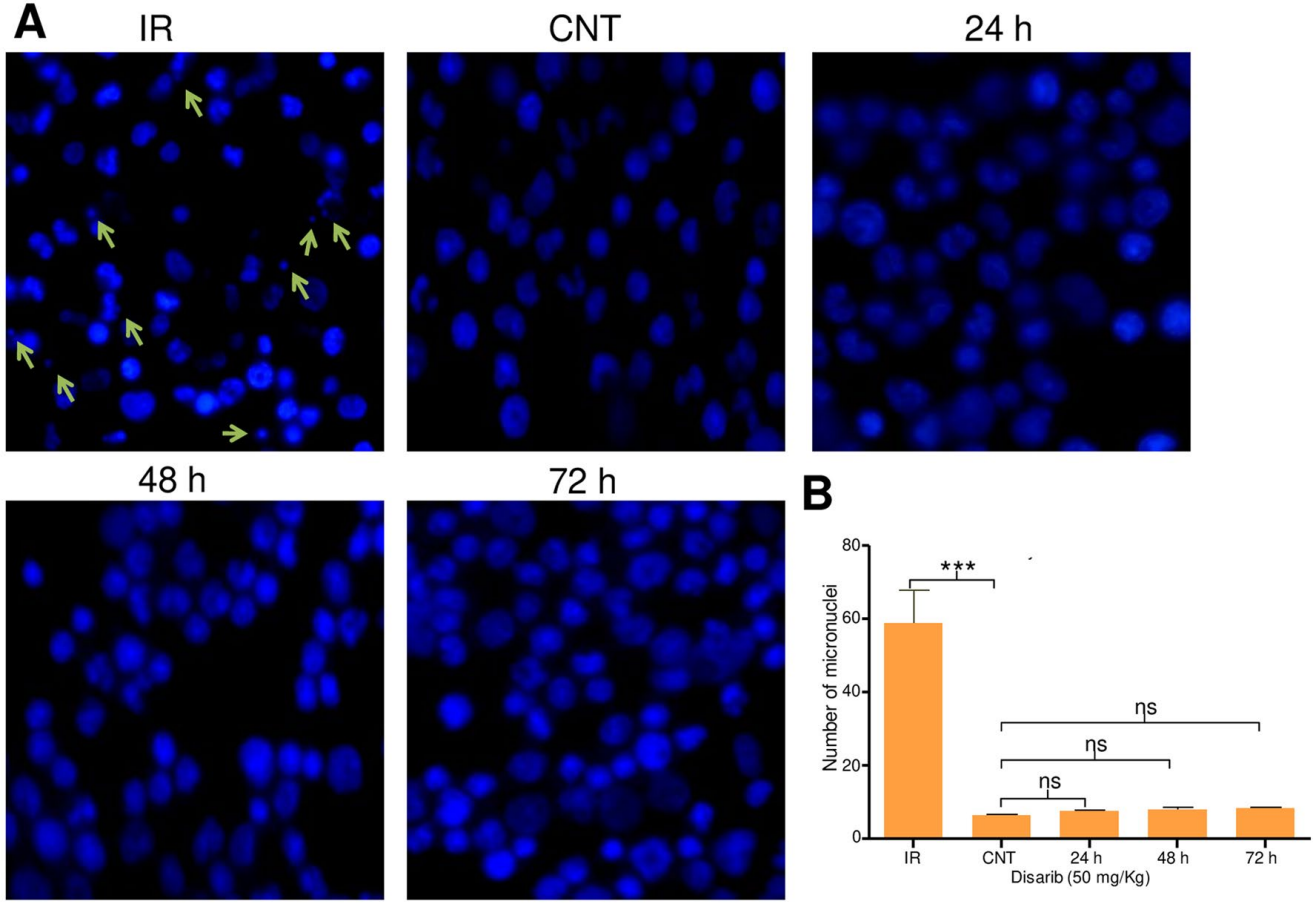
Micronucleus assay to evaluate genotoxicity of Disarib. (**A**) Representative image of vehicle control-treated and Disarib treated (24, 48 and 72 h) bone marrow cells (at 100 ×) after staining the nucleus with DAPI. In each case rats were treated with Disarib (50 mg/kg). 4 Gy IR irradiated cells served as positive control. (**B**) Bar diagram showing quantitation of micronucleus present in bone marrow cells of vehicle control (CNT), and Disarib treated animals (24, 48 and 72 h). Positive control, irradiated samples (4 Gy) were also quantitated and presented. Two animals at each time point were analyzed, and 500 cells per animal were counted for the analysis. Error bars denote mean + SEM (*ns* not significant, ***p < 0.0001).

The pharmacokinetic profile of Disarib reveals the maximum bioavailability at 24 h post Disarib oral administration. Thus, bone marrow cells were collected from rats to evaluate genotoxicity after 24 h, 48 h, and 72 h post-treatment with Disarib (50 mg/kg b. wt.). Bone marrow cells were used for micronucleus assay after fixing the cells in 2% paraformaldehyde^32–34^. Results revealed no DNA damage induced by Disarib in rats following treatment with Disarb in any of the time points investigated (Fig. 8A,B). Micronucleus formation in treated rats was comparable to that of control animals. In contrast, there was a significant increase in the number of micronuclei when rats were exposed to γ-radiation, which served as a positive control (Fig. 8A,B) for the assay. Thus in vivo genotoxicity analysis suggested that Disarib did not cause DNA damage in rats.

## Discussion

In our previous studies, we have identified a novel BCL2 specific inhibitor, Disarib^7,12,21,23^. Studies revealed that Disarib showed better efficacy when compared head to head with the FDA approved drug ABT199 in terms of cytotoxicity in cancer cell lines and tumor regression in mice^23^. When administered via oral route in mice, Disarib (50 mg/kg b. wt) showed promising anti-tumor efficacy, without any acute toxicity in mice^22^. Guidelines from CDSCO, India and FDA, USA on preclinical analysis of drug of interest suggest that systemic toxicity of any intended drug should be compared in two different rodent species (preferably mice and rats). This would help to establish linear relationship between toxicity and body surface area, so as to further determine the starting dose for future Phase I trial of Disarib. In the present study, we have performed pharmacokinetics, pharmacodynamics, and acute toxicity analysis of Disarib in another rodent species, Wistar rats, facilitating early stages of Disarib development as a potent anticancer agent.

Our studies revealed that LD_50_ of Disarib in Wistar rats is > 2000 mg/kg b. wt., which is indeed promising for further studies, and was the maximum allowed dose as per the guidelines of CDSCO, India. Importantly, this was also consistent with the results observed in mice^22^. Further, we noted that Disarib did not cause any toxicity when administered in rats, even at the highest dose.

Some of the BCL2 inhibitors failed miserably in previous studies, mainly due to dose-limiting toxicity such as neutropenia and thrombocytopenia^4,19^. Studies from the current investigation suggested no significant reduction in platelets, even when the highest dose of Disarib was orally administrated to rats. This is promising and comparable to ABT199, the only clinically approved BCL2 inhibitor in the world. In contrast, preclinical studies of BCL2 inhibitors, such as ABT737 and ABT263, showed a rapid and concentration-dependent decrease in the number of circulating platelets^20,35,36^. This was also seen when these molecules were used for clinical trials in myelodysplastic syndromes, acute myeloid leukemia and ovarian cancers. (https://www.clinicaltrials.gov/ct2/show/NCT00413114?term=obatoclax&draw=2&rank=10)^13,20,35,36^. Studies of ABT199 on the other hand showed no toxic effect on platelets in clinical trials^20^. All the other blood parameters analyzed, such as RBC, PCV, MCV, MCH, MCHC, haemoglobin, neutrophils, lymphocytes, eosinophils, monocytes, suggested that treatment with Disarib did not have any impact on the number of blood cell types.

Liver and kidney play important roles in maintaining the metabolic activity of the system. Hence, it is crucial to analyze drug-induced toxic effects on these organs. In the current study, we have analyzed the liver and kidney function by evaluating the levels of SGOT, SGPT, ALP, bilirubin, total protein, creatinine, BUN, phosphorous, and uric acid in the serum. Interestingly, we observed that treatment of Disarib did not have any ill effects on normal liver and kidney function, suggesting that Disarib did not cause toxicity to the liver and kidney. Further, histopathological analysis of tissues from organs such as liver, kidney, intestine, lung, heart, and spleen revealed no change in normal architecture and organization upon administration of Disarib than control tissues, affirming that Disarib has no deleterious effect on organ structure and histology. Similar results were also seen when Disarib was administered in mice^22^.

No detectable genotoxicity was observed in vivo when Disarib treated rat bone marrow was analyzed using micronucleus assay, indicating that Disarib did not induce DNA damage in treated animals. Therefore, these studies suggested that the administration of Disarib is safe and does not induce DNA damage.

Solubility studies revealed that Disarib was soluble in DMSO (> 75%), alcohol (> 70%), and acids (0.3 N HCl). Further, combining acidic conditions with a lower percentage of alcohol could increase the solubility in alcohol. The majority of BCL2 inhibitors, including ABT199, ABT737, and ABT263, also showed solubility in organic solvents such as DMSO and Dimethylformamide while their solubility was minimal in aqueous buffers^11,20^. ABT199 and ABT263 were also soluble in absolute ethanol^20,37^.

Disarib is stable in PBS for 6 h while in vitro plasma stability of Disarib decreased with time. Other BCL2 inhibitors, such as ABT263 and ABT737, have low plasma clearance value, which was one of the limitations in their clinical development^16^.

Pharmacokinetic studies were used to determine the bioavailability of Disarib in rat serum. The pharmacokinetics results suggested that the maximum concentration of Disarib in serum is ~ 18 µg/ml, which was seen even up to 24 h of oral administration. While ABT737 is not orally bioavailable, ABT199 and ABT263 were orally bioavailable. ABT263 showed ~ 20% of bioavailability in rats^16^. However, preclinical data of ABT263 suggested dose-limiting thrombocytopenia, which was overcome by the development of selective BCL2 inhibitor ABT199. Importantly, ABT199 also exhibited high bioavailability with half life ranging from 12 h in dogs and 2.2 h in monkey (https://www.ema.europa.eu/en/documents/assessment-report/venclyxto-epar-public-assessment-report_en.pdf)^16^. Thus, in conjunction with the previous investigation, our current study makes Disarib a potential candidate for further studies and future clinical trials.

## Methods

### Chemical synthesis

Synthesis and characterization of Disarib was reported previously^21^.

### Pharmacokinetics analysis of Disarib

Wistar rats (*Rattus norvegicus*) were administered with 50 mg/kg Disarib orally, and blood was collected by heart-puncture method following sacrifice at different time points (15 min, 30 min, 1, 2, 4, 6, 8, 10, 12, 24, 48 and 72 h) following treatment. Blood was allowed to clot, and serum was separated by centrifugation (900×*g*, 10 min) and deproteinized by adding acetonitrile. Samples for standard calibration curve were prepared by spiking 100 μM Disarib in rat plasma. The supernatant was loaded onto a C18 column, and HPLC analysis was performed in acetonitrile:water gradient, as described before^23,38^ (Shimadzu, Kyoto, Japan). Disarib specific peak were acquired at 232 nm wavelength^23^. At least two mice were sacrificed at each time point for the study. Pharmacokinetic parameters were analysed by using LabSolutions software (Shimadzu, Japan), and the values obtained were plotted with GraphPad Prism (ver5.1) software, where C is predicted concentration and t is time. Data was analysed using nonlinear regression analysis. Maximum drug plasma concentration (Cmax) and Time to reach Cmax was determind by area under the curve versus time curve. Clearance (Cl) and steady state volume of distribution (Vss) were calculated respectively by rate of drug elimination and amount of drug in the body at equilibrium condition vs steady state drug concentration in plasma.

### Disarib solubility assay

The solubility of Disarib was determined by the shake flask method, as described earlier^39^. Briefly, Disarib (100 μM) was dissolved in various solvents such as alcohol (40, 60, 80 and 100%), acid (0.1, 0.3 and 0.6 N HCl) and DMSO (25, 50, 75 and 100%). Further, solubility of Disarib was also tested in a combination of alcohol (40, 60%) and acid (0.1, 0.3 N). The suspension was vortexed 2–3 min and kept in a water bath for 5 min, as described before^25,26,39^. In each case, the samples were diluted in acetonitrile and analyzed using HPLC in acetonitrile:water gradient^27,40^. The area under the curve (AUC) was determined by using the software LabSolutions, Shimadzu (Japan). The values used for plotting in GraphPad Prism (ver 5.1) softwarewere the mean of three independent experiments.

### Determination of stability of Disarib

The stability of Disarib (100 μM) was investigated in phosphate-buffered saline (PBS) and in plasma. The blood was collected from rats by heart puncture, and plasma was separated for the study. After the addition of Disarib into plasma or PBS, the mixture was incubated at 37 °C in water bath^25,26^. The samples were then collected at different time points, 0, 2, 4, and 6 h, mixed manually with acetonitrile (1:1), and analyzed on HPLC^27,40^. The values plotted represent the mean of three independent experiments.

### Animals, grouping, and dose administration

Wistar rats, *Rattus norvegicus* (~ 175 g) were purchased from Central Animal Facility, IISc, Bangalore. All the animals were housed in polypropylene cages and kept in controlled lighting of 12 h light/dark cycle throughout the experiment. Standard pellet diet (Agro Corporation Pvt. Ltd. India) and water ad libitum have been provided to the animals. Maintenance and handling of the animals were according to the guidelines of the animal ethical committee. The experimental design and methods followed institutional guidelines and were approved by Institutional Animal Ethics Committee of Indian Institute of Science, Bangalore (Ethical committee approval No: CAF/Ethics/551/2017 and CAF/Ethics/744/2020).

All the studies were designed according to the guidelines of the Central Drug Standard Control Organization (Schedule-Y-CDSCO, Appendix III), India (https://cdsco.gov.in/opencms/opencms/en/Drugs/New-Drugs/). Single-dose toxicity analysis was performed in male Wistar rats. Animals were segregated in groups of five, and Disarib was administered using oral gavage (100, 1000, 2000 mg/kg b. wt.). Carboxymethyl cellulose, used for preparing Disarib, served as vehicle control to feed control animals. Mortality, general appearance, and behavior were initially observed for 24 h. Animals were further observed for 14 days and were further subjected to toxicity analysis.

### Determination of LD_50_

The median lethal dose was determined in male Wistar rats. Adult male rats of comparable body weight (~ 175 g) were divided into four groups (n = 5). Disarib was orally administered after making solution using carboxymethyl cellulose (100, 1000, 2000 mg/kg), and lethality was evaluated over 14 days after treatment. Carboxymethyl cellulose treated animals served as vehicle control.

### General observation

General cage-side examination of the treated and control animals were performed every day during the experiment. General behavior, appearance, skin, hairs, secretion (lachrymation), and death were evaluated daily, according to the guidelines of CDSCO, India^41,42^.

Body weight was recorded on every 4th day from the date of administration of Disarib up to 32 days. Food and water consumption were monitored every day from the date of administration of the compound. During the present study, on every alternate days ~ 250 g food pellet and ~ 500 ml water were given to each cage (control and treatment groups). There were no significant differences observed in food and water consumption of animals in each cage compared to control.

### Haematological analysis

After 14 days of Disarib treatment, two animals from each group were sacrificed using CO_2_ asphyxiation, and blood was collected in EDTA-coated vials by heart puncture. Red blood cells (RBC), white blood cells (WBC), platelet counts (PLT), packed cell volume (PCV), hemoglobin (HGB), mean cell volume (MCV), mean corpuscular hemoglobin (MCH), mean corpuscular hemoglobin concentration (MCHC), lymphocytes, neutrophils, eosinophils and monocytes were analyzed as described before^28,31,43^. Analysis of blood samples was performed at Rohana Veterinary Diagnostic Lab, Bangalore, India.

### Serum biochemistry

After 14 days of Disarib administration, two animals were sacrificed using CO_2_ asphyxiation from each group, and blood was collected in tubes without using anticoagulant and centrifuged (900×*g*, 10 min). Serum glutamic pyruvic transaminase (SGPT), serum glutamic oxaloacetic transaminase (SGOT), alkaline phosphatase (ALP), albumin, total protein, bilirubin, blood urea nitrogen (BUN), creatinine, uric acid, and phosphorous were analyzed as described earlier^28,29^. Analysis of serum samples was performed at Rohana Veterinary Diagnostic Lab, Bangalore, India.

### Histology

At the end of the experimental period (14 days), two animals from each group were sacrificed following CO_2_ asphyxiation. Liver, kidney, intestine, spleen, lungs, and heart were collected, fixed in 4% paraformaldehyde (PFA) and processed for histology, as described before^44–46^. For histological studies, tissues from rats treated with the highest dose (2000 mg/kg) and the vehicle control group were selected. Paraffin blocks were prepared and sections were taken using a rotary microtome (Leica Biosystems, Buffalo Grove, IL, USA) with a thickness of 5 µm. Following deparaffination, sections were stained with hematoxylin and eosin (H&E), mounted in DPX, and imaged under a bright-field microscope (Carl Zeiss AxioVision, Oberkochen, Germany).

### Genotoxicity evaluation

To evaluate genotoxicity caused by Disarib at the effective dose, micronucleus assay was performed^32–34^. Wistar rats (n = 6) were orally administered with Disarib (50 mg/kg), and bone marrow cells were collected after 24, 48, and 72 h post-treatment. Bone marrow cells were flushed out in 1X PBS containing EDTA, centrifuged, fixed (2% PFA) and stained with DAPI. Cells exposed to γ-irradiation (4 Gy) served as a positive control for the assay and were imaged using a fluorescence microscope (Nikon, Tokyo, Japan). At least 500 cells were counted from each animal for each time point, and data was plotted and represented as a bar diagram^47^.

### HPLC analysis

The samples were analyzed by HPLC using the Shimadzu HPLC system. LC was carried out on the C18 analytical column. The mobile phase was the gradient of acetonitrile and water. Disarib specific peaks were detected at 232 nm. The injection volume was 20 μl, and the flow rate was 1.2 ml/min. The standard curves were constructed by plotting the peak area vs. concentration of Disarib^40^.

### Statistical analysis

Statistical analysis was performed in GraphPad Prism (ver5.1) software (GraphPad Software, Inc.) using one way ANOVA. In case of analysis of body weight and blood parameters, non-parametric Kruskal–Wallis test was performed. Analysis of effects of genotoxicity between positive control and treated samples were analysed using one way ANOVA (post hoc Dunnett test).

### Ethical approval

This is to confirm that the study decribed in the current manuscript was carried out in compliance with the ARRIVE guidelines. All the procedures, experimental design and methods performed in the study were in accordance with institutional guidelines and were approved by Institutional Animal Ethics Committee, Indian Institute of Science, Bangalore. Ethical committee approval No: CAF/Ethics/551/2017 and CAF/Ethics/744/2020.
